# Benjamin (Ben) Steinberg, MD FRCPsych

**DOI:** 10.1192/bjb.2017.24

**Published:** 2018-04

**Authors:** J. Guy Edwards

Formerly Consultant Psychiatrist, Knowle Hospital, Fareham and Royal South Hants Hospital, Southampton, UK


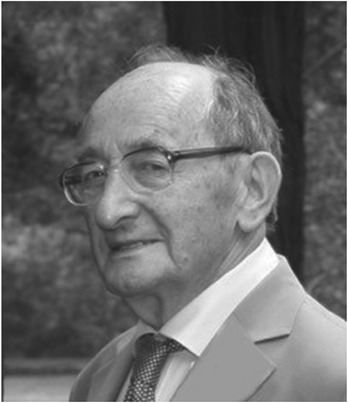


Ben Steinberg, who died recently at the age of 93 years, was regarded as one of Hampshire's most popular and well-respected psychiatrists. He had an encyclopaedic knowledge of many subjects, including psychiatry, world affairs, history, literature and the theatre, and was a most engaging conversationalist. He could debate controversies in a robust but sensitive way, and was a great listener. He had an unrelenting capacity to care. In his later years, he worked voluntarily for a charity providing emotional, legal and financial support for refugees and asylum seekers in Southampton and Winchester.

He served as chairman of the Medical Committee, was a member of the Management Committee and chairman of the Psychiatric Rehabilitation Committee at Knowle Hospital, was chairman of the Wessex Psychotherapy Society, and was also a member of the Southampton and District Community Health Council and chairman of its Mental Health Group. He taught under- and post-graduate students and assisted in a number of research projects.^1^^,^^2^

He was an active member of the Labour Party and was elected chairman of the Romsey and Southampton North Branch and chairman of the Southampton and District Fabian Society.

Ben was born into a Jewish family in Latvia. In 1925, his family moved to Belfast where his father became an assistant Rabbi. He graduated in medicine from Queen's University, Belfast, in 1946 and obtained his MD in 1957. Following his preregistration training, he served in the Royal Army Medical Corps for 2 years and then undertook his psychiatric training (1949–1956). He was appointed Senior Hospital Medical Officer in Cardiff in 1956 and consultant at Knowle Hospital in 1958. After retiring he worked as clinical assistant in the Rehabilitation and Long-Stay Units at Knowle and Southampton for a further 9 years.

He and his late wife, Phyllis (a nurse from the Republic of Ireland), showered hospitality on his colleagues and many generations of trainees and their partners. Many overseas trainees showed their appreciation decades after returning to their native countries.

Ben remained youthful until near the end. His clinical skills, intellectual prowess, sense of duty, concern for patients and loyalty to friends and colleagues will remain as monuments to his memory. He will be sadly missed by all who knew him, but most of all by his family, to whom he was deeply devoted – his three children (David, Judith and Helen), ten grandchildren and two great grandchildren.
